# Dental implants in rehabilitation for cleft orthognathic: A clinical study

**DOI:** 10.6026/973206300221440

**Published:** 2026-03-31

**Authors:** Samir Mansuri, K Jyothirmayee, Namratha Lakshmi Agnihotri, Subbaraju Tetali, Akhilesh Prathap, M Athira, Heena Dixit Tiwari, Rahul Tiwari

**Affiliations:** 1 Department of Oral and Maxillofacial surgery, Al Kuwait Hospital, Dubai, United Arab Emirates; 2 Department Of Oral And Maxillofacial Surgery, Government Dental College And Hospital, Afzalgunj, Hyderabad, Telangana, India; 3 Department of Prosthodontics, Consultant Prosthodontist, Bhubaneswar, Odisha, India; 4 Department of Oral and Maxillofacial Surgery, Nimra Institute of Dental Sciences, Nimra Nagar, Jupudi (V), Ibrahimpatnam (M), Krishna, Andhra Pradesh 521456, India; 5 Department of Oral and Maxillofacial Surgery, Pushpagiri College of Dental Sciences, Thiruvalla, Kerala, India; 6 Department of Prosthodontics, Educare Institute of Dental Sciences, Malappuram, Kerala, India; 7 Consultant, Blood Cell, Commissionerate of Health and Family Welfare, Government of Telangana, Hyderabad, Telangana, India; 8 Department of Oral and Maxillofacial Surgery, RKDF Dental College and Research Centre, Sarvepalli Radhakrishnan University, Bhopal, Madhya Pradesh, India

**Keywords:** Dental implants, alveolar cleft graft, orthognathic surgery, prosthodontic rehabilitation, cleft lip and palate

## Abstract

Implant-based rehabilitation in patients with cleft lip and palate following orthognathic correction remains challenging due to altered bone quality, scarred soft tissues and concerns regard long-term stability and prosthetic complications. Therefore, it is of interest to evaluate clinically the survival rate of implants and the success rate of prosthetics in patients with clefts who underwent staged rehabilitation post-orthognathic treatment. This took place after standardized monitoring for follow-up and peri-implant conditions. Very high short-term implant survival was noted with complications limited to cases that needed regrafting or those requiring complicated prosthetic contours within the cleft segment. Substantial enhancement in functional recovery and patient-reported satisfaction took place when design considerations for prosthetics gave priority to hygiene access plus management of soft tissue. Implant rehabilitation becomes predictable among appropriately selected patients having cleft-orthognathic conditions when there is integration between surgical planning and prosthodontic implementation.

## Background:

Cleft lip and palate patients often have hypodontia in the cleft area, reduced alveolar dimension and a difficult set of occlusal aspects that persists into adult life. Implant restorations in grafted cleft sites have very good survival in them. This means that implants can be successful for a long time when bone and soft tissue conditions are well maintained [1, 2]. However, implant failures and prosthetic complications are still a clinical concern. Particularly when cleft morphology, periodontal restrictions of adjacent teeth and compliance considerations all intersect [4]. Literature reviews describe bone-grafted cleft sites with implant survival rates over 90% and note variable esthetic outcomes and emphasis on patient-reported outcomes [4, 5]. Newer grafting approaches, including cortical block augmentation for deficient cleft sites, may expand candidacy for implant placement and reduce reliance on compromised prosthetic alternatives [5]. Therefore, it is of interest to evaluate implant-based rehabilitation effects in cleft patients managed within an orthognathic pathway.

## Materials and Methods:

Current prospective clinical cohort research was conducted in a tertiary cleft-orthognathic service between January 2022 and December 2025. Adult patients (≥18 years) with repaired cleft lip and/or palate who needed orthognathic correction (Le Fort I with or without bilateral sagittal split osteotomy) and subsequent replacement of at least one missing tooth in the cleft segment were incorporated. Subjects with syndromic clefts, active uncontrolled periodontitis, heavy tobacco use, or uncontrolled systemic diseases known to impair bone healing were excluded. All participants underwent orthognathic correction using a digital planning workflow incorporating cone-beam computed tomography (CBCT) and intraoral scans. Preparation of the cleft-segment implant site followed one of two approaches: implant placement into previously grafted cleft bone when bone volume was adequate, or staged augmentation (including particulate grafting or cortical block grafting when indicated) followed by delayed implant placement. Prosthetic rehabilitation was performed using screw-retained restorations whenever feasible, with emergence profiles designed to facilitate hygiene access and provide soft-tissue support. The primary outcome measure was implant survival at 24 months after prosthetic loading. Secondary outcomes consisted of peri-implant marginal bone level change as assessed by standardized radiographs, prosthetic complications rate and patient-reported satisfaction (on a 10-point Likert scale). Descriptive statistics were used and chi-square or Fisher's exact test for categorical variables, t-test for independent samples or Mann-Whitney U test for continuous variables were made with statistical significance at α=0.05.

## Results:

Forty patients with cleft (average age 24.9) attended the rehabilitation path; most were affected by unilateral CLP. Many underwent bimaxillary orthognathic surgery, indicating complex sagittal and vertical corrections. A total of 52 implants were planned and approximately one third of sites required further augmentation prior to implantation. Thus, reflecting cleft-segment bone deficits. Implant position was placed generally in the first postoperative year following orthognathic surgery to moderate occlusal stabilization and establish final prosthetic space Table 1 (see PDF). At 24 months, mean peri-implant marginal bone change was 0.84 ± 0.46 mm and prosthetic complications occurred in 8 cases (15.4% of implant-supported restorations), most commonly screw loosening that was managed chairside without the need for prosthesis remanufacture Table 2 (see PDF).

## Discussion: 

The cleft orthognathic rehabilitation needs to be aligned with the skeletal correction, scarred soft tissues and prosthetic space. The current results-favorable early implant survival and satisfactory peri-implant bone maintenance-are in line with a contemporary concept focusing on digital-based, integrated planning and sequenced site conditioning. The emerging cleft orthognathic literature highlights that the trend for more accurate planning is becoming increasingly reliant on virtual workflows with an increasing scoping evidence base pointing to greater uptake of VSP/CAD-CAM in cleft orthognathics, together with reporting trends toward enhanced predictability and standardized outcome tracking [6]. Systematic review of orthognathic outcomes in cleft populations also emphasizes inconsistently high relapse and reoperation risk, advocating for postponing permanent implant prosthodontics until skeletal stability is achieved [7]. Relapse is one of the main factors threatening long-term occlusal stability for cleft orthognathic cases. This latter reason is currently under clinical discussion and also the need for long-term postoperative follow-up is being emphasized (especially in complicated cases of maxillary advancement) [8]. Twenty-year follow-up has indeed shown some risk factors for reoperation after cleft orthognathic surgery and have established that some patients will return with the need for a second skeletal revision, at which time function may be compromised in terms of prosthesis position, occlusal contacts and prosthetic life [9]. Three-dimensional stability studies following bimaxillary osteotomy also agree that the potential for measurable hard tissue changes postoperatively exists and thus implant placement should be based on a protocol of individualized stability evaluation rather than according to arbitrary time intervals [10]. Patients with more severe maxillary hypoplasia, poor graft quality or unfavourable cleft-segment anatomy may be candidates for other forms of anchorage.

A patient-customized implant technique (IPC guideline) has been retrospectively analyzed and found to be feasible for difficult cleft rehabilitation when conventional implants or grafting options are not available but generalization of these findings and cost-effectiveness depend on further validation [11]. The choice of prosthesis is also important: cleft patients usually receive hygiene-compatible interfaces and removable/maintainable superstructures. A clinical series with single-tooth CAD/CAM superstructures for cleft patients indicates that implant-supported bar/telescope designs may be able to lower the complication rate and facilitate long- term care within a cleft-unit [12]. Zygomatic implants provide another alternative when posterior maxillary bone is insufficient or serial grafting is not desired. A narrative review has recently suggested additional well-defined referral and outcome on behalf of zygomatic implants in cleft care, advocating their use as graft-avoiding approaches in limited cases [13]. Case-level literatures still highlights technical feasibility in patients with a cleft, but also suggest the demand for uniform definition of complications and multidisciplinary follow-up [14]. Last, basal implant principles have been concluded to be a viable option in cleft cases but evidence is scarce and should not replace conventional planning if grafted alveolar sites are treatable [15].

## Conclusion:

Predictable implant-based rehabilitation of the edentulous cleft-orthognathic patient can be accomplished with staged planning that balances skeletal stability, site augmentation requirements and prosthetic spatial control. The risk of complications is increased in anatomically compromised cleft segments and pathways predisposed to relapse or reoperation. An inter-disciplinary approach using digital workflow and hygienic prosthetic design will lead to enhanced functional and patient-based outcomes.

## Advancement to knowledge:

This prospective clinical study provides contemporary (2020-2025-aligned) outcome data demonstrating that implant placement following orthognathic correction in cleft patients can achieve high short-term survival with acceptable marginal bone stability, while quantifying augmentation needs, prosthetic complication patterns and patient-reported satisfaction within an integrated cleft-orthognathic pathway.
